# Polyvinylidene Fluoride-Based Nanowire-Imprinted Membranes with High Flux for Efficient and Selective Separation of Artemisinin/Artemether

**DOI:** 10.3390/molecules29163868

**Published:** 2024-08-15

**Authors:** Minjia Meng, Jiajia Ren, Chuanxun Zhang, Wanqi Du, Jixiang Wang

**Affiliations:** School of Chemistry and Chemical Engineering, Jiangsu University, Zhenjiang 212013, China; j2733827931@163.com (J.R.); zcx622820@163.com (C.Z.); dwq11292@126.com (W.D.)

**Keywords:** artemisinin, selective separation, blend membrane, molecular imprinting technology

## Abstract

A traditional phase transformation method is commonly used to prepare molecular imprinting membranes for selective separation. However, traditional molecularly imprinted polymers are mostly micron-sized particles, and the imprinting sites in their membrane are easily embedded, leading to a reduced adsorption capacity and decreased selectivity. In this study, an ultra-long nanowire with a diameter of about 15 nm was synthesized for the separation of artemisinin (ART), and its adsorption capacity was as high as 198.29 mg g^−1^ after imprinting polymerization. Molecular imprinting membranes were prepared, using polyvinylidene fluoride (PVDF), polyethersulfone (PES), and polysulfone (PSF) as the membrane matrix, for comparison. The average membrane pore size of PVDF-MIM was about 480 nm, and PVDF-MIM had the highest adsorption capacity (69 mg g^−1^) for ART. The optimal flow rate for PVDF-MIM’s dynamic adsorption of ART was 7 mL min^−1^. Under this optimal flow rate, selectivity experiments were carried out to obtain the separation factor of PVDF-MIM (α = 8.37), which was much higher than the corresponding values of PES-MIM and PSF-MIM. In addition, the hydrophobicity and low flux of PES-MIM and PSF-MIM lead to higher non-specific adsorption. The hydrophobicity of PVDF-MIM is lower than that of PES-MIM and PSF-MIM, which greatly reduces the non-specific adsorption of the membrane, thus increasing the selectivity of the membranes. Therefore, the effective density of the imprinting sites in the pores and the membrane structure are the main factors determining the efficient separation of molecularly imprinted membranes.

## 1. Introduction

Artemisinin (ART) is a sesquiterpene lactone compound with a unique chemical structure, derived from Artemisia annua [1]. It is soluble in chloroform, acetone, ethyl acetate, ethanol, and other organic solvents, slightly soluble in cold petroleum ether, and almost insoluble in water. Due to its special peroxy group, ART is thermally unstable and easily decomposed by moisture, heat, and reducing substances. Since its discovery, ART has become the most important drug for treating malaria, one of the most widespread and destructive infectious diseases, due to its specific activation and drug target mechanisms. In addition, according to modern research, ART shows strong pharmacological antibacterial anti-inflammatory effects, as well as effects on immune regulation and anti-tissue fibrosis [1,2,3,4]. Artemether (ARE), also known as methyl-reduced artemisinin, one of the main derivatives of ART, is recommended by the World Health Organization as a first-line drug for the treatment of falciparum malaria [5]. From 1976 to 1978, it was proved, by animal experiments and clinical trials of ARE injections, that the antimalarial effect of ARE was several times higher than that of ART, adverse reactions were fewer, the drug’s effect was faster, and the recurrence rate was lower [4,5]. The synthesis of ARE is generally based on using ART as the raw material [6]. In the reaction process of synthesizing ARE with ART as the raw material, the yield is easily affected by the amount of reducing agent used, reaction temperature, reaction time, and other factors, but in order to ensure the purity and medicinal value of ART, it is very important to effectively separate the final ART (excess reactant) and ARE (product). The most commonly used separation methods are microwave extraction [7], ultrasonic wave extraction [8], organic solvent extraction [9], supercritical CO_2_ extraction [10], recrystallization [11], frying, and chromatography [12]. However, each of these methods has various drawbacks and cannot fully meet the drug’s separation requirements. Therefore, it is very important to propose effective ways to improve the selective separation efficiency of ART and ARE that can be widely used in practical production.

A molecular imprinting membrane [13,14,15] is a new type of separation membrane that combines molecular imprinting technology [16] and membrane separation technology [17,18], which has high selectivity and a molecule-specific recognition ability and has the advantages of molecular imprinting and the characteristics of a separation membrane, with great potential in separation engineering. Molecular imprinting membranes are usually prepared by a phase inversion method [16,19]. The traditional phase inversion method involves dissolving a specific amount of template molecules, along with one or more functional polymers, into an appropriate solvent. This solution is then spread onto a support body and transformed from a liquid to a solid state through a suitable process to prepare a molecular imprinting membrane. Although this method is simple to perform, can be easily run continuously, and operates under mild conditions, it fails to achieve effective selective separation. This is because the solvent’s swelling during the traditional phase conversion process alters the size of the imprinting sites, reducing their ability to recognize specific substances. Consequently, the selectivity of the molecular imprinting membrane generated is significantly diminished. The primary polymerization method, coating method, and electrochemical method used have a series of disadvantages, such as low membrane flux, a weak separation performance, complicated steps, and large experimental requirements. Therefore, this study has enhanced the traditional phase inversion method, retaining its inherent advantages while also significantly improving its selective separation efficiency of ART and ARE [20].

To address the bottlenecks seen in membrane fabrication using the phase inversion method, we plan to first construct molecularly imprinted materials and then blend them with a casting solution to prepare molecularly imprinted membranes [21,22]. The resulting imprinted membranes will not experience a reduction in selectivity at their imprinted sites due to solvent swelling. However, traditional molecularly imprinted polymer particles, despite having effective recognition sites and small imprinting sizes, tend to have their imprinting sites embedded by the casting solution during the phase inversion process. This leads to a reduction in effective recognition sites and significantly decreases their separation efficiency. MnO_2_ nanowires have been shown to be an ideal choice for creating membrane materials because of their excellent mechanical properties, chemical stability, environmental compatibility, and abundant availability. Additionally, ultra-long manganese dioxide nanowires are a preferential candidate for synthesizing molecularly imprinted materials, as they provide a rich density of recognition sites per unit volume. Ultra-long MnO_2_-imprinted nanowires can significantly mitigate the drawbacks of the low separation efficiency caused by the embedding of traditional particle-type imprinted polymers during their phase inversion [23,24]. This improvement is primarily due to two reasons: firstly, the length of the nanowires reduces the likelihood of their complete embedding, allowing them to be more readily exposed within the membrane pores; secondly, MnO_2_ itself is a hydrophilic material with numerous polar groups on its surface [25], which tend to migrate towards water molecules during phase inversion, thereby enhancing the distribution of MnO_2_-imprinted nanowires within the pores.

In this study, MnO_2_-based molecularly imprinted nanowires were synthetized first, and then three varied imprinted membranes with the same added imprinted nanowires, using polyvinylidene fluoride (PVDF), polyethersulfone (PES), and polysulfone (PSF) as their membrane matrices, were prepared via the phase inversion method. The preparation and characterization of the imprinted nanowires and imprinted membranes were studied. Then, their static and dynamic adsorption performance toward ART was explored in detail. The effect of the membrane’s structure (porosity, flux, and hydrophilicity) on its ART/ARE separation performance was also studied.

## 2. Experimental

### 2.1. Materials

Potassium sulfate (K_2_SO_4_), potassium persulfate (K_2_S_2_O_8_), manganese sulfate monohydrate (MnSO_4_·H_2_O), acetonitrile (CH_3_CN), acetic acid (C_2_H_4_O_2_), DIMETHYL SULFOXIDE-D6 (DMSO), hydrochloric acid (HCl), and ethanol (C_2_H_5_OH) were purchased from National Pharmaceutical Chemical Reagent Co., Ltd. (Shanghai, China). Acrylamide (AM), ethylene glycol dimethacrylate (EGDMA), Azobisisobutyronitrile (AIBN), artemisinin (ART), artemether (ARE), artesunate (ARU), and Tetrafluoroethylene (PVDF, M_W_ = 110 kDa) were obtained from the Aladdin Co., Ltd. (Shanghai, China).

### 2.2. Synthetic Experiments

#### 2.2.1. Synthesis of MnO_2_ Nanowires

A mixture of K_2_SO_4_ (37.87 mmol, 6.6 g), K_2_S_2_O_8_ (75.3 mmol, 20.5 g), and MnSO_4_·H_2_O (43.71 mmol, 6.6 g) was ground, transferred to a hydrothermal synthesis kettle, had 60 mL of deionized water added, and was stirred to form a saturated solution. Then, it was reacted at 250 °C for 4 days. The resulting reactants were washed with deionized water at 60 °C until the color changed from black to brown and then washed with acetonitrile several times. After the washed reactants were violently stirred at 3000 r/min and 25 °C for 6.0 h, pure and evenly distributed manganese dioxide nanowires were obtained and transferred to the centrifuge tube for use [18].

#### 2.2.2. Synthesis of Imprinted Polymers

The dummy molecules ARU (0.1 mmol, 0.0384 g) and AM (0.4 mmol, 0.0284 g) were dissolved in 60 mL of acetonitrile in 100 mL round-bottom flasks and then refrigerated for 2 h to obtain self-assembled prepolymers of ARU and AM. The MnO_2_ nanowires (0.34 mmol, 30 mg), EGDMA (2.0 mmol, 0.3964 g), and AIBN (0.18 mmol, 0.03 g) were fully mixed in a solution and then high-purity N_2_ was introduced for 10 min to remove O_2_. Finally, the reaction was carried out for 16 h and at 140 rpm in 65 °C water bath oscillations. After polymerization, the product was separated by centrifugation, dried with a deionized water wash sample, and eluted in a soxhlet extractor with a methanol–acetic acid mixture (*v:v* = 9:1), until the pseudo-template molecule (ARU) was undetectable at 237 nm using a UV photometer. Finally, after several washes with anhydrous ethanol and drying for 12 h in a vacuum drying chamber (45 °C), MnO_2_ nanowire molecular imprinting polymers (MIPs) were obtained. Non-imprinted polymers (NIPs) based on the MnO_2_ nanowires were prepared via the same experimental steps but without adding the pseudo-template molecule ARU.

#### 2.2.3. Synthesis of MnO_2_-Nanowire-Blended Imprinting Membrane

**Figure 1:**
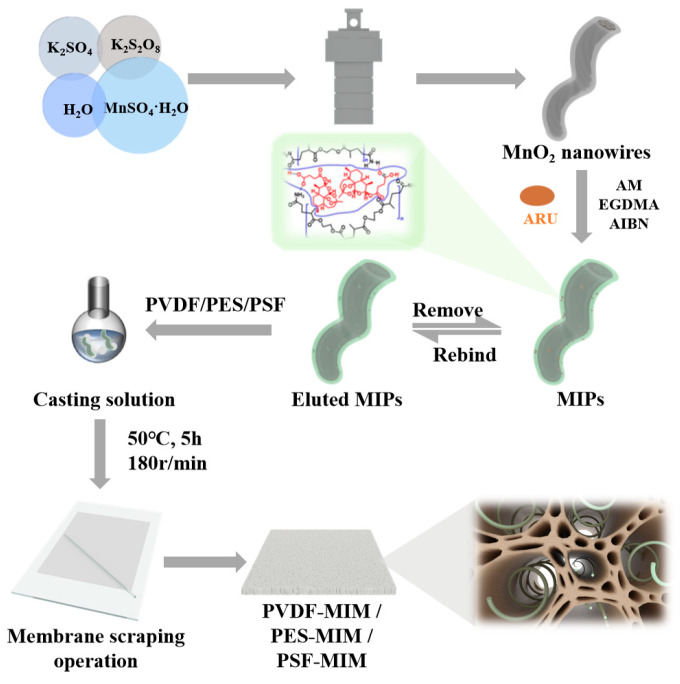
Flow chart of the preparation of molecularly imprinted membranes.

A total of 0.42 g of PVDF powder and 0.01 g of MIPs were dissolved in 5.58 g of DMSO, and continuous magnetic stirring at 180 r/min for 5 h at 50 °C was carried out to form a uniform casting membrane solution. We poured the casting solution onto the glass plate, scraped it lightly with a glass rod, put it into deionized water immediately, and replaced the DMSO completely. PVDF-MIM was stored in deionized water for further testing. Then, PES-MIM and PSF-MIM were obtained via the same procedure, except that PVDF powder was replaced by PES or PSF powder. The synthetic constituents of these membranes are listed in Table 1. Blank membranes without MIPs were created following the same steps, and were marked as PVDF, PES, and PSF. The flow chart of their preparation is shown in Figure 1.

### 2.3. Flux Test

PVDF-MIM, PES-MIM, PSF-MIM, PVDF, PES, and PSF were installed on a self-made dead-end filter. The effective membrane area was 3.14 cm^2^ and the working pressure was 0.1 MPa. The feed liquid was continuously passed through the membrane on the dead-end filtering device. The flux (*J*, L m^2^ h^−1^) should be repeated at least 3 times, as determined by Formula (1) [26].

(1) $$J=\frac{V}{st}$$

In this equation, *J* (L m^2^ h^−1^) is the membrane flux, *V* (L) is the volume of permeation, *t* (h) is the time required for permeation, and *s* (m^2^) is the effective area of the membrane.

### 2.4. Adsorption Experiment

#### 2.4.1. MIPs/NIPs Adsorption

Three different concentrations (20, 40, 60 mg L^−1^) of ART ethanol solution were prepared; 10 mL of the prepared solution was taken and 5 mg of MIPs/NIPs was added. The concentrations were measured by HPLC and their adsorption capacity was calculated according to Formula (2).

(2) $$q_{e}=\frac{(C_{0}-C_{e})V}{M}$$

where *q_e_* (mg g^−1^) is the adsorption capacity, *C*_0_ (mg L^−1^) is the initial concentration, *C_e_* (mg L^−1^) is the equilibrium concentration of the solution, *V* (L) is the volume of the solvent, and *M* (g) is the mass of the adsorbent.

#### 2.4.2. Optimal Concentration

We prepared ART ethanol solutions of 40, 80, 100, 200, 300, 500 mg L^−1^ and put PVDF-MIM, PES-MIM, PSF-MIM, PVDF, PES, and PSF into 10 mL of each of the prepared solutions. Then, we left them to adsorb for 2 h in a 25 °C water bath with vibration. The solutions’ concentration was measured by HPLC and the membranes’ adsorption capacity was calculated according to Formula (2).

#### 2.4.3. Adsorption Kinetics

An ART ethanol solution (100 mg L^−1^) was prepared, and 10 mL of it was added to PVDF-MIM, PES-MIM, and PSF-MIM and adsorbed in a 25 °C water bath, with vibration, for 20, 40, 60, 80, 100, 120, 140, 160, and 180 min, respectively. The solutions’ concentration was measured by HPLC and the membranes’ adsorption capacity was calculated according to Formula (2).

In order to better study the adsorption kinetics of ART on PVDF-MIM, PES-MIM, and PSF-MIM, pseudo-first-order and pseudo-second-order models were used to study the adsorption rate and rate control mechanism of the whole adsorption process, which are shown in Formulas (3) and (4) [27].

(3) $$q_{t}=q_{e}-q_{e}e^{-k_{1}t}$$

(4) $$q_{t}=\frac{k_{2}q_{e}^{2}t}{1+k_{2}q_{e}t}$$

In these equations, *q_t_* (mg g^−1^) and *q_e_* (mg g^−1^) are the adsorption amounts of the adsorbents at time *t* and equilibrium time, respectively. *k*_1_ (min^−1^) and *k*_2_ (g mg^−1^ min^−1^) are pseudo-first-order and pseudo-second-order model rate constants, respectively.

#### 2.4.4. Sorption Isotherm

We prepared ART ethanol solutions of 20, 100, 200, 300, 400, and 500 mg L^−1^ and put PVDF-MIM, PES-MIM, and PSF-MIM into 10 mL of each of the prepared solutions. Then, we left them to adsorb for 2 h in a 25 °C water bath with vibration. The solutions’ concentration was measured by HPLC and the membranes’ adsorption capacity was calculated according to Formula (2).

Langmuir and Freundlich models were used to investigate the isothermal adsorption behavior of ART on PVDF-MIM, PES-MIMm and PSF-MIM. The fitting coefficient (*R*^2^) was obtained by analyzing the fitting data to determine a suitable model for the isothermal adsorption experiment. 

The Langmuir model [28] is expressed by Formula (5):

(5) $$q_{e}=\frac{q_{m}K_{L}C_{e}}{1+K_{L}C_{e}}$$

Here, *C_e_* (mg L^−1^) is the ART concentration at equilibrium, *q_e_* (mg g^−1^) and *q_m_* (mg g^−1^) are the equilibrium adsorption capacity and maximum adsorption capacity of MIPs and NIPs for ART, respectively, and *K_L_* (L mg^−1^) is a constant related to the affinity between the adsorbent and the absorbent.

The Freundlich model [29] is expressed by Formula (6):

(6) $$q_{e}=K_{F}C_{E}^{1/n}$$

*K_F_* (mg^−1^) and n are empirical constants representing the Freundlich adsorption isotherm constant and heterogeneity coefficient, respectively. When the value of *n* is greater than 1, it is preferentially adsorbed.

#### 2.4.5. Adsorption Thermodynamics

PVDF-MIM, PES-MIM, and PSF-MIM containing 10 mg of MIPs were placed in an ART ethanol solution (100, 200, 300, 400, 500 mg L^−1^) at 15 °C and 25 °C, respectively, and reacted for 2 h. After adsorption, the concentration was measured by HPLC, and the adsorption capacity of the membranes was calculated by Formula (2).

The thermodynamic parameters include Δ*G* (kJ mol^−1^), Δ*S* (kJ mol^−1^ K^−1^), and Δ*H* (kJ mol^−1^), which can be calculated from Equations (7)–(9).

(7) $$\Delta G=-RTlnK_{d}$$

(8) ΔG=ΔH+TΔS

(9) $$lnK_{d}=-\frac{\Delta H}{RT}+\frac{\Delta G}{R}$$

where *R* (8.314 J mol^−1^ K^−1^) is the universal gas constant, *T(K)* is the temperature, *K_d_* (L g^−1^) is the equilibrium constant, and *K_d_* (L g^−1^) = *q_m_* × *K_L_* of the Langmuir isothermal model.

#### 2.4.6. Adsorption Selectivity

In the experiments in this section, a 300 mg L^−1^ ART and ARE two-component solution was configured, and the sample membrane and 10 mL of the two-component solution were added to a centrifuge tube. Then, it was placed in a constant-temperature water bath oscillator at 25 °C; after adsorption for 2 h, the concentration of ART and ARE in the solution was determined. The concentration was measured by HPLC and the equilibrium adsorption capacity of ART and ARE in the solution can be calculated using Formula (2).

The distribution coefficient (*K_d_*) and selectivity coefficient (*α*) of PVDF-MIM, PES-MIM, and PSF-MIM for ART and ARE were calculated by Formulas (10) and (11).

(10) $$k_{d}=\frac{q_{e}}{C_{e}}$$

In this formula, *q_e_* (mg g^−1^) is the adsorption capacity of ART or ARE and *C_e_* (mg L^−1^) is the equilibrium concentration of ART or ARE.

(11) $$\alpha =\frac{K_{d\left(ART\right)}}{K_{d\left(ARE\right)}}$$

In this formula, *K_d_*_(*ARE*)_ and *K_d_*_(*ART*)_ represent the partition coefficients of the ART and ARE in the solution, respectively.

#### 2.4.7. Dynamic Adsorption

Dynamic cross-flow experiments were carried out on a flat film tester (TYLG-18); the PVDF-MIM, PES-MIM, and PSF-MIM (4.9 cm in diameter) were correctly placed on the sample stage of a membrane module (21.23 cm^2^ effective filtration area). At 25 °C and 0.1 MPa, 250 mL of 500 mg L^−1^ ART ethanol was circulated at flow rates of 3, 5, 7, and 10 mL min^−1^ in the sample bed and material tank. Furthermore, it was sampled at 0, 5, 10, 30, 60, 120, and 180 min. The solution’s concentration was measured by HPLC and the adsorption capacity of the materials was calculated according to Formula (2).

#### 2.4.8. HPLC

The adsorption concentration was measured by an HPLC-Agilent-1260 (Agilent, Santa Clara, CA, USA). A Capcell PAK AQ C18 column (250 mm × 4.6 mm, 3 μm) was used, the mobile phase was acetonitrile–acetic acid solution = 60:40 (*v:v*, pH = 4.5), the flow rate was 1 mL/min, the column temperature was 30 °C, the length of the column was 213 nm, and the volume of the injection was 10 μL.

### 2.5. Characterization Method

The X-ray diffractometer (XRD) used was Germany’s BRUKER-D8-ADVANCE (Bruker, Ettlingen, Germany). The working voltage and current were set at 40 kV and 30 mA. The scanning mode was continuous scanning, the step length was 0.02°/step, the scanning range was 10~80°, and the scanning time was 15 min. A membrane with a diameter of about 0.5 cm was taken and adhered to the glass sheet. The test was then run.

The field emission scanning electron microscope (SEM) used was the S-4800 model of the Hitachi Corporation of Japan (Tokyo, Japan). The MnO_2_ nanowires/MIPs/NIPs/membrane surface/membrane cross-section were glued to the sample tray with conductive adhesive, and then gold-sprayed and tested.

The transmission electron microscope (TEM) used was the JEM-2100 model of Japan Electronics Co., LTD. (JEOL, Tokyo, Japan). A small amount of the MnO_2_ nanowires/MIPs/NIPs was added to a centrifuge tube, with ethanol added, stirred and ultrasonicated, and a small amount of liquid was absorbed with an eyedropper and dropped on an electron microscope copper net, which could be tested after drying.

The Fourier infrared spectrometer (FT-IR) used was the Nicolet Nexus 470 model of Nicolet (Appleton, WI, USA). A small amount of potassium bromide was ground, manually pressed, and tested, which was then used to eliminate the sample’s background. We added 1 mg of MIPs/NIPs to a mortar, added 100 mg of KBr for full grinding, took a small amount of evenly mixed powder, and tested it on a manual tablet. We took a small piece on the prepared membrane, placed the instrument in the corresponding position, and tested it. 

The water contact angle (WCA) test instrument adopted to press the water sample to be tested was the German Klyusch DSA25S (Kruss Scientific Instruments, Hamburg, Germany). The sample to be tested was compressed and a small amount of deionized water was absorbed into a syringe and slowly dropped on the sample while waiting for the water droplets to stabilize and recording the value of the contact angle.

The Accelerated Surface Area and Porosimetry System (BET) used was a Belsorp-Mini, a Micrometer from Quantachrome Instruments, which was used to measure the pore size of the membrane.

The Thermal Gravimetric Analyzer (TG) used was the German Nike TG 209 F3 (Berlin, Germany).

## 3. Results and Discussions

### 3.1. Characterization of Nanowire-Based Molecular Imprinting Polymers

#### 3.1.1. SEM, TEM, and Contact Angle Test

**Figure 2:**
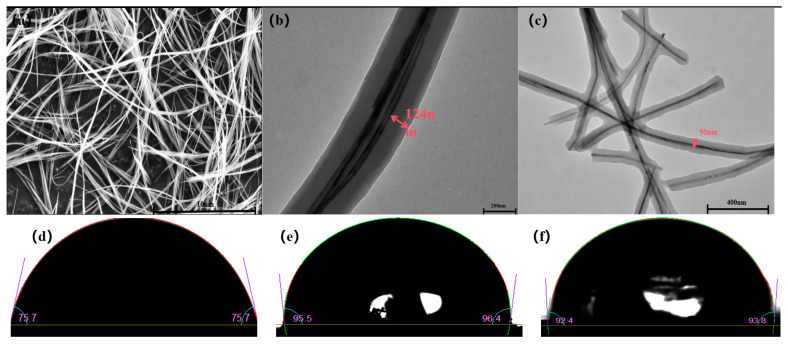
MnO_2_ nanowire scans (**a**). Transmission maps of the polymerization layer thickness of MIPs (**b**) and NIPs (**c**). The contact angle images of the pretreated powder, measured using a static drop mode, of MnO_2_ (**d**), MIPs (**e**), and NIPs (**f**).

The diameter of the MnO_2_ nanowires (Figure 2a) is about 15 nm and they have an extremely long aspect ratio, which has the potential to increase the number of effective recognition sites. After polymerization, the thickness of the MIPs’ and NIPs’ imprinting layers are about 124 nm and 50 nm, respectively, as shown in Figure 2b,c. The imprinted layer thickness of the MIPs is greater than that of the NIPs, which may be due to the hydrogen bonding force between the template molecule and the functional monomer, which forms a cavity during polymerization, making the polymer larger in volume, whereas no template molecule is involved in the NIPs synthesis, and as such they will not produce a cavity, which leads to a small-size polymer. The contact angles of the MnO_2_ nanowire, MIPs, and NIPs are 75.7°, 96.4°, and 93.8°, respectively (Figure 2d–f), which indicate that the nanowire is hydrophilic and becomes hydrophobic after imprinting, which demonstrates the successful synthesis of nanowire-based imprinting polymers.

#### 3.1.2. FT-IR, TG, and Adsorption Experiment

**Figure 3:**
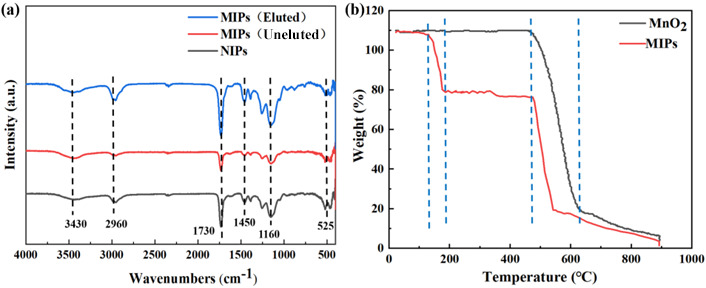
FT-IR spectra of eluted MIPs, uneluted MIPs, and NIPs (**a**). TG curves of MnO_2_ and MIPs in nitrogen atmosphere with heating rate of 20 °C min^−1^ (**b**).

**Figure 4:**
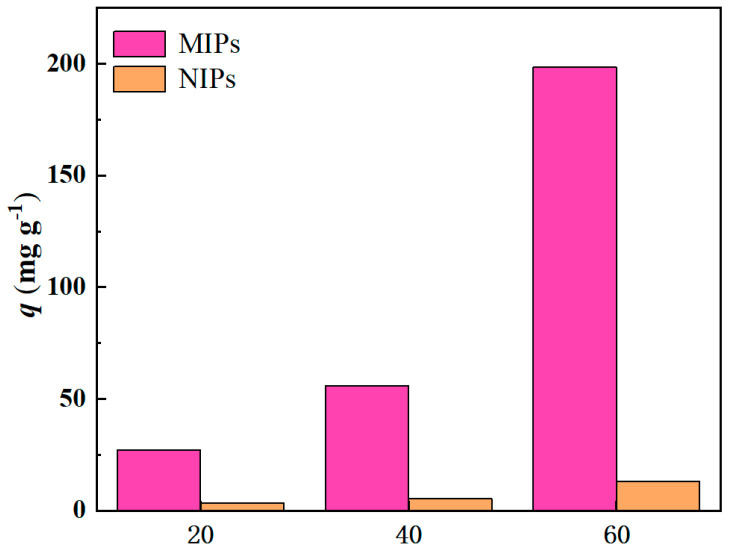
The adsorption capacity of MIPs and NIPs at three different concentrations.

The FT-IR spectra of uneluted MIPs, eluted MIPs, and NIPs are shown in Figure 3a. The characteristic peak of Mn-O in the MnO_2_ nanowires appears at 525 cm^−1^. After imprinting polymerization, the characteristic peaks of the MIPs and NIPs at 1160 cm^−1^ were attributed to the C-O-C stretching vibration of EGDMA used in the imprinting polymerization, while the peak at 1730 cm^−1^ represented the C=O characteristic peak in EGDMA versus AM [30]. The peaks at 2960 cm^−1^ and 1450 cm^−1^ represent the tensile vibration of C-H in -CH_3_ and the asymmetric in-plane bending vibration of C-H in -CH_3_, respectively. The wide and weak absorption peaks at 3430 cm^−1^ may be the tensile vibrations of the hydrogen bonds formed by N-H bonds. Through a thermogravimetric analysis, as shown in Figure 3b, thermogravimetric tests were carried out on the successfully prepared MnO_2_ and MIPs at a 20 °C min^−1^ heating rate in a N_2_ atmosphere. MnO_2_ begins to decompose at 420 °C, with a weight loss of 88%. MIPs lose 0.57% of their mass at around 100 °C, which is some of the oligomers on the surface of the MIPs. Then, decomposition began at 120~180 °C, where the mass loss was 19%, which was the decomposition of the imprinting layer on the surface of the MIPs. Finally, at 420 °C, MnO_2_ begins to decompose. This proves that we have successfully prepared MIPs. The adsorption data on MIPs and NIPs in ART ethanol solutions with concentrations of 20, 40, and 60 mg L^−1^, respectively, are shown in Figure 4. The adsorption capacities of the MIPs at the three concentrations are 27.93, 56.08, and 198.29 mg g^−1^, respectively, much higher than that of the NIPs at the three concentrations, which are 3.83, 6.14, and 13.51 mg g^−1^. At the same time, the adsorption capacities of the MIPs and NIPs increased with the increase in concentration, which was in accordance with the law of adsorption thermodynamics, which indicates that the synthesized MIPs had an excellent performance and laid a foundation for the preparation of highly selective imprinted membranes.

### 3.2. Characterization of Membranes

#### 3.2.1. SEM and Physical Drawings

**Figure 5:**
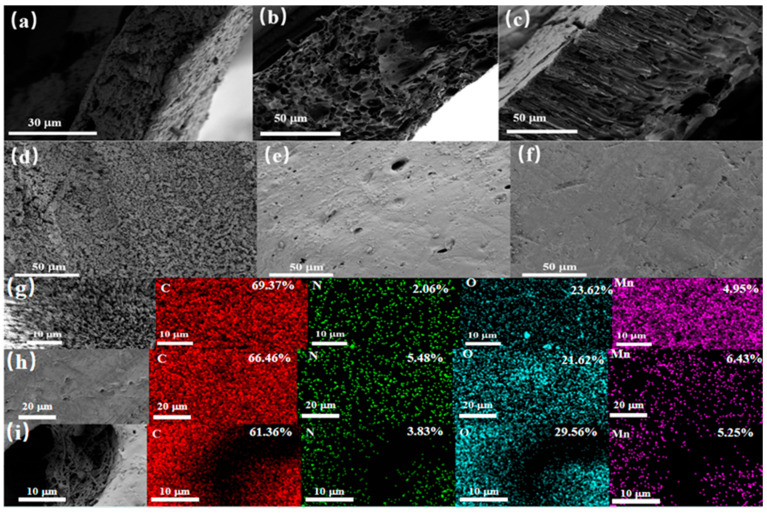
SEM of PVDF-MIM, PES-MIM, and PSF-MIM sections (**a**–**c**). SEM of PVDF-MIM, PES-MIM, and PSF-MIM (**d**–**f**). Corresponding element mapping of PVDF-MIM, PES-MIM, and PSF-MIM (**g**–**i**).

**Figure 6:**
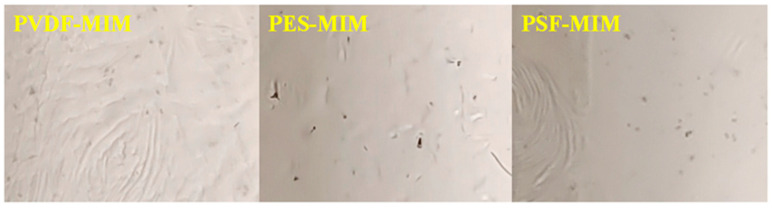
Digital photos of PVDF-MIM, PES-MIM, and PSF-MIM.

The morphology of PVDF-MIM, PES-MIM, and PSF-MIM was characterized by SEM, as shown in Figure 5. Figure 5a–c are the cross-sectional images of PVDF-MIM, PES-MIM, and PSF-MIM, respectively. Figure 5d–f are the surface scanning images of PVDF-MIM, PES-MIM, and PSF-MIM. It can be seen that the aperture distribution of PVDF-MIM is more uniform and denser. Figure 5g–i are the corresponding element maps of PVDF-MIM, PES-MIM, and PSF-MIM, respectively. C, N, O, and Mn were well distributed on PVDF-MIM, PES-MIM, and PSF-MIM’s surfaces, with corresponding atomic ratios of 69.37% (C), 2.06% (N), 23.62% (O), and 4.95% (Mn); 66.46% (C), 5.48% (N), 21.62% (O), and 6.43% (Mn); 61.36% (C), 3.83% (N), 29.56% (O), and 5.25% (Mn), respectively. Their atomic contents are very similar, which shows that the modified phase inversion method is universal and the MIPs are distributed uniformly in the three kinds of membranes, which are comparable. Digital photos of PVDF-MIM, PES-MIM, and PSF-MIM are shown in Figure 6.

#### 3.2.2. XRD and FT-IR Studies

**Figure 7:**
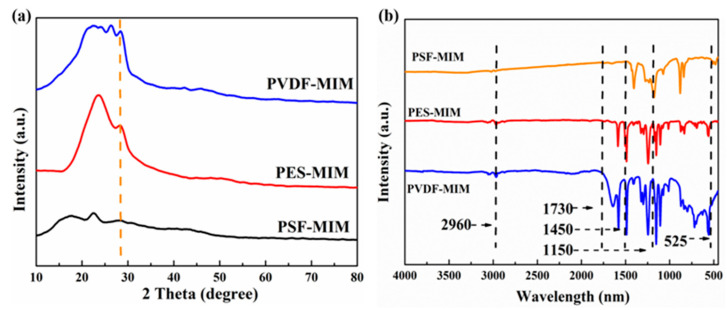
XRD spectra (**a**) and FT-IR spectra (**b**) of PVDF-MIM, PES-MIM, and PSF-MIM.

The XRD patterns of PVDF-MIM, PES-MIM, and PSF-MIM are shown in Figure 7a. The common crystalline forms of PVDF include *α* phase (usually two main characteristic peaks between 25 and 45°), *β* phase (whose characteristic peaks are mainly concentrated between 20 and 30°), and *γ* phase (whose main characteristic peaks appear in the range of 20~25°). According to Figure 7a, the PVDF membrane has a wide peak between 20 and 27°, indicating that this PVDF has a *β* phase form. This gives the PVDF a high degree of symmetry, giving the PVDF excellent permeability and flexibility [31]. As the MnO_2_ nanowires are embedded in the imprinted layer, their detection signal is weak. At 2*θ* = 28.610°, PVDF-MIM, PES-MIM, and PSF-MIM all have characteristic peaks, which is consistent with the main characteristic peak of MnO_2_. The FT-IR spectra of the PVDF-MIM, PES-MIM, and PSF-MIM membranes are shown in Figure 7b, and at 525 cm^−1^ the characteristic peaks of the Mn-O in the MnO_2_ nanowires are visible. At 1730 cm^−1^, PVDF-MIM, PES-MIM, and PSF-MIM all have characteristic peaks which are attributed to the C=O tensile vibration in EGDMA. The peaks at 2960 cm^−1^ and 1450 cm^−1^ represent the tensile vibration of C-H in -CH_3_ and the in-plane asymmetric bending vibration of C-H in -CH_3_, respectively. The results indicated that PVDF-MIM, PES-MIM, and PSF-MIM were successfully prepared.

#### 3.2.3. Analysis of Membrane Pore Size, Membrane Flux, and Contact Angle

**Figure 8:**
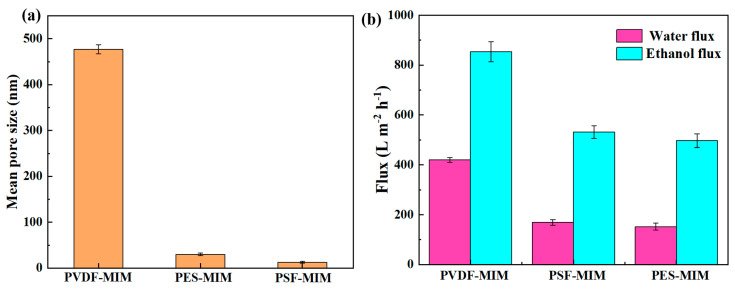
Average pore size (**a**) and membrane flux (**b**) of PVDF-MIM, PES-MIM, and PSF-MIM blended membranes.

**Figure 9:**
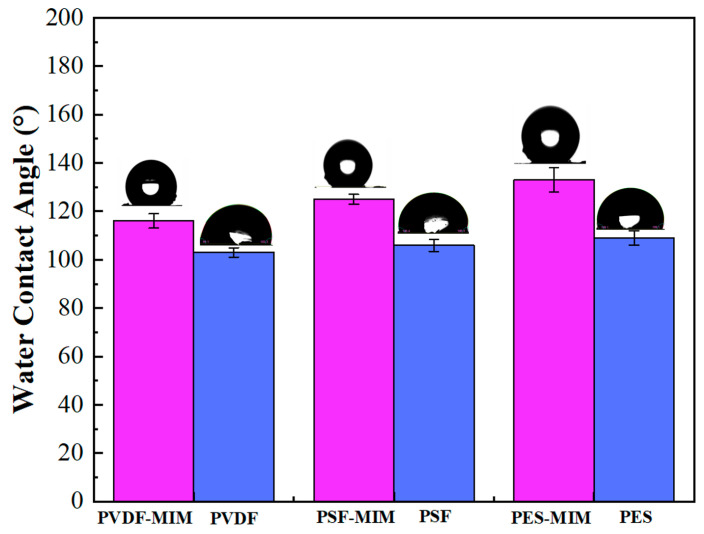
Water contact angle of PVDF-MIM, PVDF, PES-MIM, PES, PSF-MIM, and PSF.

Water flux and ethanol flux measurements and the characterization of the membrane pore sizes of PVDF-MIM, PES-MIM, and PSF-MIM are shown in Figure 8. Figure 8a shows that the average pore size of PVDF-MIM is 477 nm, which also far exceeds that of PES-MIM and PSF-MIM, so PVDF-MIM has a better membrane flux. As shown in Figure 8b, the water flux of PVDF-MIM (420 L m^−2^ h^−1^) was much larger than that of PES-MIM (169 L m^−2^ h^−1^) and PSF-MIM (152 L m^−2^ h^−1^); the ethanol flux of PVDF-MIM (854 L m^−2^ h^−1^) was also much larger than that of PES-MIM (497 L m^−2^ h^−1^) and PSF-MIM (532 L m^−2^ h^−1^), which is also consistent with the characterization of their mean pore sizes in Figure 8a. The larger the pore size, the higher the membrane flux. The ethanol flux is higher than the water flux, because PVDF, PSF, and PES have strong hydrophobicity. As can be seen from Figure 9, the water contact angles of PVDF, PSF, PES, PVDF-MIM, PSF-MIM, and PES-MIM are all larger than 90°; furthermore, their hydrophobicity was increased after adding the hydrophobic MIPs.

### 3.3. Adsorption Performance

#### 3.3.1. The Best Adsorption Concentration

**Figure 10:**
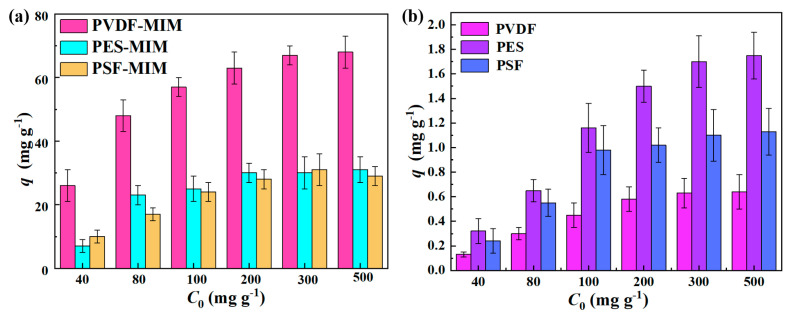
Comparison of the adsorption capacity of ART on PVDF-MIM, PES-MIM, and PSF-MIM (**a**) and comparison of the adsorption capacity of ART on PVDF, PES, and PSF (**b**).

The adsorption capacities of PVDF-MIM, PES-MIM, and PSF-MIM under different concentrations of ART ethanol solution are shown in Figure 10a. The adsorption capacity of PVDF-MIM was the highest, reaching 69 mg g^−1^; its adsorption capacity is more than two times that of PES-MIM and PSF-MIM (about 30 mg g^−1^). The equilibrium adsorption capacity of PVDF-MIM, PES-MIM, and PSF-MIM increased with the increase in the ART concentration. When the concentration of ART was 200~500 mg L^−1^, the adsorption saturation of each membrane was reached. PVDF, PES, and PSF are hydrophobic, and ART is also hydrophobic, so these membranes adsorb a small amount of ART due to this non-specific adsorption, even without the addition of MIPs, as shown in Figure 10b.

#### 3.3.2. Adsorption Kinetics

**Figure 11:**
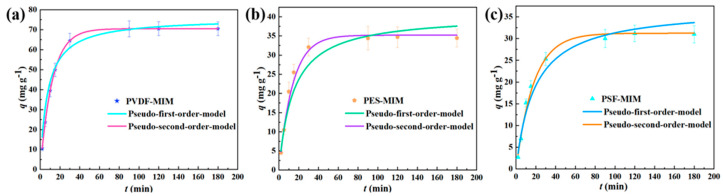
The kinetic model fitting of PVDF-MIM (**a**), PSE-MIM (**b**), and PSF-MIM (**c**) to the adsorption of ART: pseudo-first-order model and pseudo-second-order model.

In order to better simulate the adsorption behavior of PVDF-MIM, PES-MIM, and PSF-MIM, we carried out adsorption kinetics experiments. The instantaneous adsorption capacities of PVDF-MIM, PES-MIM, and PSF-MIM at various times are shown in Figure 11. From the adsorption process of three samples, we can conclude that PVDF-MIM reaches its adsorption equilibrium at 90 min, and the adsorption amount reaches 70.5 mg g^−1^. PES-MIM and PSF-MIM reach their adsorption equilibrium at 100 and 120 min, their adsorption capacities were 34.45 and 31.0 mg g^−1^, respectively. This indicates that there are more effective recognition sites in PVDF-MIM and that the adsorption capacity of PVDF-MIM is increased.

The adsorption of ART onto PVDF-MIM, PES-MIM, and PSF-MIM can be divided into two processes: fast recognition adsorption and slow recognition adsorption. At the initial stage of adsorption, the PVDF-MIM, PES-MIM, and PSF-MIM blended membranes all contain a large number of imprinted sites, and the ART molecules can bind rapidly until most of the imprinted sites are occupied, and then the rate at which ART molecules bind to the imprinted holes slows gradually until equilibrium is reached.

The correlation coefficients (*R*^2^) of the pseudo-first-order models of PVDF-MIM, PES-MIM, and PSF-MIM were 0.83, 0.80, and 0.81, respectively, which are larger than the corresponding coefficients (*R*^2^ = 0.99) of the pseudo-second-order models. The data are shown in Table 2. Therefore, the above data show that the adsorption of ART onto PVDF-MIM, PES-MIM, and PSF-MIM is more in accordance with the pseudo-second-order kinetic model. Therefore, we can better describe the whole adsorption process by using the pseudo-second-order kinetic model.

#### 3.3.3. Sorption Isotherm

**Figure 12:**
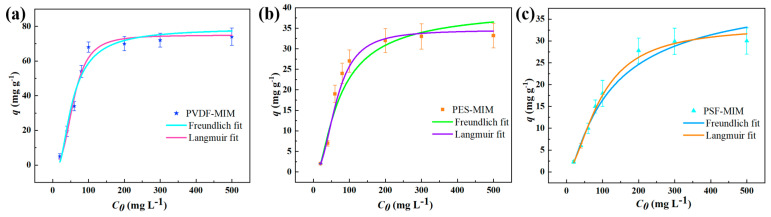
Adsorption isotherms of ART onto PVDF-MIM (**a**), PES-MIM (**b**), and PSF-MIM (**c**).

The adsorption capacities of PVDF-MIM, PES-MIM, and PSF-MIM in ethanol solutions containing different concentrations of ART are shown in Figure 12. The equilibrium adsorption capacity of PVDF-MIM, PES-MIM, and PSF-MIM increased with the increasing the concentration of ART until it reached 300 mg L^−1^, where PVDF-MIM, PES-MIM, and PSF-MIM reached their maximum adsorption capacity. However, from the graph we can intuitively see that the maximum adsorption capacity of PVDF-MIM is much higher than that of PSE-MIM and PSF-MIM. According to two kinds of models, Langmuir and Freundlich, the number obtained by fitting is shown in Table 3. The correlation coefficients of the Langmuir models for PVDF-MIM, PES-MIM, and PSF-MIM were all 0.99, much higher than those of the Freundlich models for PVDF-MIM, PES-MIM, and PSF-MIM (*R*^2^ = 0.96, 0.95, 0.97). Langmuir model was fitted to obtain the *q_m,cal_* values (74.90, 34.56, 33.41 mg g^−1^) of PVDF-MIM, PES-MIM, and PSF-MIM, which were closer to our experimental values *q_e,exp_*. The Freundlich model’s fitting shows that the n of PVDF-MIM, PES-MIM, and PSF-MIM are all greater than 1, indicating that the adsorption reaction takes precedence.

#### 3.3.4. Thermodynamics of Adsorption

**Figure 13:**
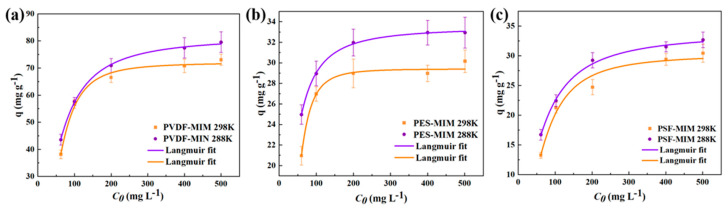
Adsorption isotherms of ART onto PVDF-MIM (**a**), PES-MIMm (**b**), and PSF-MIM (**c**) at different temperatures.

To study the thermodynamic functions of PVDF-MIM, PES-MIM, and PSF-MIM, the adsorption capacities of PVDF-MIM, PES-MIM, and PSF-MIM in ART ethanol solutions of different concentrations were calculated at 15 °C and 25 °C. As shown in Figure 13, the amount of ART adsorbed by PVDF-MIM, PES-MIM, and PSF-MIM increased with an increase in the initial concentration of the ART ethanol solution. With increasing temperature, the adsorption capacity of PVDF-MIM, PES-MIM, and PSF-MIM for ART decreased. Through a fitting analysis of the Langmuir model, the correlation coefficients *R*^2^ = 0.9875, 0.9872, and 0.9961 were obtained. The relevant thermodynamic parameters are shown in Table 4; it can be seen that in the process of the adsorption of ART by PVDF-MIM, PES-MIM, and PSF-MIM, Δ*G* < 0, which indicates that these three adsorption processes are spontaneous reactions. Δ*H* < 0, which indicates that ART can be absorbed by PVDF-MIM, PES-MIM, and PSF-MIM and that its adsorption on PES-MIM and PSF-MIM is an exothermic reaction, so their adsorption capacity will decrease with the increase in temperature. Δ*S* < 0, indicating that the disturbance of the reaction system is reduced during the adsorption process.

### 3.4. Adsorption Selectivity

**Figure 14:**
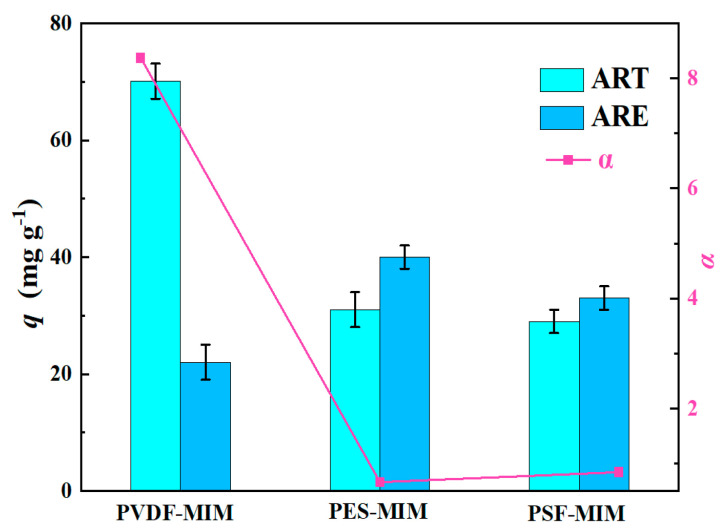
Selective adsorption of ART and ARE by PVDF-MIM, PES-MIM, and PSF-MIM.

We selected ARE as a competitive adsorbent to study the selective recognition of ART by PVDF-MIM, PES-MIM, and PSF-MIM. From Figure 14, it can be seen that PVDF-MIM has a high adsorption capacity and that its separation factor (*α* = 8.37) is much higher than that of PES-MIM (*α* = 0.66) and PSF-MIM (*α* = 0.84). The data is shown in Table 5. The adsorption capacities of PES-MIM and PSF-MIM for ART and ARE were similar, and their separation effect was not good.

### 3.5. Dynamic Adsorption

**Figure 15:**
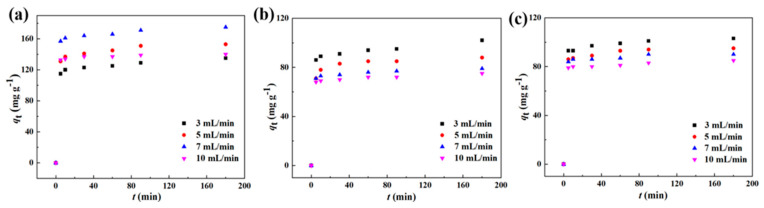
Dynamic separation and adsorption of ART by PVDF-MIM (**a**), PES-MIM (**b**), and PSF-MIM (**c**) at 3.0, 5.0, 7.0, and 10.0 mL.

In order to obtain the best flow rate at the highest adsorption capacity, dynamic separation experiments of PVDF-MIM, PES-MIM, and PSF-MIM were carried out. As shown in Figure 15, the adsorption capacity of PVDF-MIM, PES-MIM, and PSF-MIM increased with time at the same flow rate, and the adsorption rate was very fast in the first 5 min. As shown in Figure 15a–c, the optimal flow rate for PVDF-MIM was 7 mL min^−1^, while for PES-MIM and PSF-MIM it was 3 mL min^−1^. Different membranes have different optimal flow rates, which are determined by their own characteristics. In the previous characterization, it was seen that the pore size and strength of each membrane are different.

## 4. Conclusions

PVDF-MIM has larger membrane pore size and higher membrane flux, which means that more of its imprinted sites adsorb target molecules; the static adsorption results also showed that PVDF-MIM has the highest adsorption capacity (69.0 mg g^−1^) for ART, which is more than twice as high as that of PES-MIM (31.0 mg g^−1^) and PSF-MIM (29.0 mg g^−1^). The isothermal adsorption results show that the adsorption process is a spontaneous exothermic process, in accordance with the Langmuir adsorption model, and its adsorption kinetics conform to the pseudo-second-order kinetic equation. The difference in *k*_2_ and *q_e_* in the different membranes is due to their different adsorption velocities, caused by their pore size, strength, hydrophobicity, and so on. The equilibrium adsorption capacity of PVDF-MIM is much higher than that of PES-MIM and PSF-MIM. This also shows that PVDF has the best adsorption performance for ART.

The optimal flow rate of PVDF-MIM was determined to be 7 mL min^−1^, and that of PES-MIM and PSF-MIM was 3 mL min^−1^; the separation factor of PVDF-MIM (*α* = 8.37) was much higher than that of PES-MIM (*α* = 0.66) and PSF-MIM (*α* = 0.84). This is attributed to the hydrophilicity and high throughput of PVDF. The data fully show that the membrane matrix is one of the most important factors affecting the efficient selective separation of ART.

## Figures and Tables

**Table 1:** Parameters of membrane preparation process.

Blended Imprinted Membrane	*m*/g	*m*/g	*m*/g	*m*/g	*m*/g
	PVDF	PSF	PES	DMSO	MIPs
**PVDF-MIM**	0.42	-	-	5.58	0.01
**PSF-MIM**	-	0.6	-	5.40	0.01
**PES-MIM**	-	-	0.6	5.40	0.01

**Table 2:** Dynamic parameters of pseudo-first-order and pseudo-second-order equations.

Membrane	Pseudo-First-Order Kinetic Equation				Pseudo-Second-Order Kinetic Equation		
	*q_e,cal_* (mg g^−1^)	*k*_1_ (min^−1^)	*R^2^*	*q_e,exp_* (mg g^−1^)	*q_e,cal_* (mg g^−1^)	*k*_2_ (g mg^−1^ min^−1^)	*R* ^2^
**PVDF-MIM**	75.85	0.0081	0.83	70.50	70.49	0.0821	0.99
**PES-MIM**	40.56	0.0017	0.80	34.60	35.10	0.0723	0.99
**PSF-MIM**	37.19	0.0014	0.81	31.20	31.23	0.0560	0.99

**Table 3:** Langmuir and Freundlich isotherm constants of ART’s adsorption onto PVDF-MIM, PES-MIM, and PSF-MIM.

Models	Parameter	PVDF-MIM	PES-MIM	PSF-MIM
**Langmuir model**	***R*** **^2^**	0.99	0.99	0.99
	***K_L_*** **(L mg^−^^1^*)***	6.69 × 10^−6^	4.42 × 10^−5^	3.94 × 10^−4^
	***q_m,cal_*** **(mg g** **^−^** **^1^** **)**	74.90	34.56	33.41
**Freundich model**	***R^2^***	0.96	0.95	0.97
	***K_F_*** **(mg g^−1^)**	78.87	40.02	43.66
	***n***	1.86	1.31	1.1

**Table 4:** Thermodynamic parameters of PVDF-MIM, PES-MIM, and PSF-MIM.

Membrane	∆H	∆S	∆G (kJ mol^−1^)	
	(kJ mol^−1^)	(kJ mol^−1^ k^−1^)	15 °C	25 °C
**PVDF-MIM**	−46.72 ± 2.10	−0.17 ± 0.03	−2.81 ± 0.3	−1.32 ± 0.20
**PES-MIM**	−2.53 ± 0.32	−0.012 ± 0.001	−0.85 ± 0.25	−0.85 ± 0.15
**PSF-MIM**	−9.20 ± 1.5	−0.036 ± 0.004	−1.31 ± 0.17	−0.98 ± 0.10

**Table 5:** Selective adsorption parameters of PVDF-MIM, PES-MIM, and PSF-MIM.

	*q* (mg g^−1^)		*K_d_* (L g^−1^)		*α*
Membrane	ART	ARE	ARE	ARE	
**PVDF-MIM**	70.1 ± 5.5	22.0 ± 1.8	2.344 ± 0.30	0.28 ± 0.02	8.37 ± 1.01
**PES-MIM**	30.3 ± 2.1	39.8 ± 2.7	0.435 ± 0.09	0.661 ± 0.04	0.66 ± 0.10
**PSF-MIM**	29.2 ± 2.5	33.1 ± 2.5	0.41 ± 0.05	0.49 ± 0.03	0.84 ± 0.09

## Data Availability

The data are contained within the article.

## References

[1] Laminou I.M., Issa I., Adehossi E., Maman K., Jackou H., Coulibaly E., Tohon Z.B., Ahmed J., Sanoussi E., Koko D. (2024). Therapeutic efficacy and tolerability of artemether-lumefantrine for uncomplicated Plasmodium falciparum malaria in Nige. Malar. J..

[2] Bonepally K.R., Takahashi N., Matsuoka N., Koi H., Mizoguchi H., Hiruma T., Ochiai K., Suzuki S., Yamagishi Y., Oikawa H. (2020). Rapid and systematic exploration of chemical space relevant to Artemisinins: An-ti-malarial Activities of Skeletally Diversified Tetracyclic Peroxides and 6-Aza-artemisinins. J. Org. Chem..

[3] Crespo-Ortiz M.P., Wei M.Q. (2011). Antitumor activity of artemisinin and its derivatives: From a well-known antimalarial agent to a potential anticancer drug. J. Biomed. Biotechnol..

[4] Khanal P. (2021). Antimalarial and anticancer properties of artesunate and other artemisinins: Current development. Monatshefte Fuer Chemie/Chem. Mon..

[5] Esu E.B., Effa E.E., Opie O.N., Meremikwu M.M. (2020). Artemether for severe malaria. Emerg. Rev. De La Soc. Esp. De Med. De Emerg..

[6] Wang M., Liu X., Zheng X., Luo Y., Gao Y., Chen H. (2024). Synthesis of Artemisinin G from Artemisinin via Photocatalysis. Eur. J. Org. Chem..

[7] Fernandez-Pastor I., Fernandez-Hernandez A., Perez-Criado S., Rivas F., Martinez A., Garcia-Granados A., Parra A. (2017). Microwave-assisted extraction versus Soxhlet extraction to determine triterpene acids in olive skins. J. Sep. Sci..

[8] Komartin R.S., Stroescu M., Chira N., Stan R., Stoica-Guzun A. (2021). Optimization of oil extraction from *Lallemantia iberica* seeds using ultra-sound-assisted extraction. J. Food Meas. Charact..

[9] Lou X., Zhu A., Wang H., Wu J., Zhou L., Long F. (2016). Direct and ultrasensitive optofluidic-based immunosensing assay of aflatoxin M1 in dairy products using organic solvent extraction. Anal. Chim. Acta.

[10] Gadkari P.V., Balaraman M. (2017). Mass transfer and kinetic modelling of supercritical CO_2_ extraction of fresh tea leaves (*Camellia sinensis* L). Braz. J. Chem. Eng..

[11] Zu Y.G., Wang L., Zhao X., Li Y., Wu W., Zu C., Huang Y., Wu M., Feng Z. (2016). Purification of *Ginkgo biloba* extract by antisolvent recrystallization. Chem. Eng. Technol..

[12] Uno T., Niioka T., Hayakari M., Sugawara K., Tateishi T. (2007). Simultaneous determination of warfarin enantiomers and its metabolite in human plasma by column-switching high-performance liquid chromatography with chiral separation. Ther. Drug Monit..

[13] Masakazu Y., Kalsang T., Ştefan-Ovidiu D. (2016). Molecularly imprinted membranes: Past, present, and future. Chem. Rev..

[14] Yang H., Liu H.-B., Tang Z.-S., Qiu Z.-D., Zhu H.-X., Song Z.-X., Jia A.-L. (2021). Synthesis, performance, and application of molecularly imprinted membranes: A review. J. Environ. Chem. Eng..

[15] Chen J., Wei M., Meng M. (2023). Advanced Development of Molecularly Imprinted Membranes for Selective Separation. Molecules.

[16] Wang Z., Yu S., Wang H., Wang J., Xiao S. (2023). Research progress and application prospects of molecularly imprinted membrane technology: A review. Mater. Res. Innov..

[17] Bi Y., Dong J., Zhou Y., Zhang M., Chen X., Zhang Y. (2024). Application of membrane separation technology in the purification of pharmaceutical components. Prep. Biochem. Biotechnol..

[18] Ma R., Li J., Zeng P., Duan L., Dong J., Ma Y., Yang L. (2024). The application of membrane separation technology in the pharmaceutical industry. Membranes.

[19] Yu C., Song J., Yan Y., Gao J., Xing W., Meng M., Yan Y., Ma Z., Wu Y. (2022). A “graphdiyne-like” anti-fouling TBBPA molecularly imprinted membrane synthesized based on the delayed phase inversion method: A concomitant permeability and selectivity. J. Membr. Sci..

[20] Zhang Y., Tan X., Liu X., Li C., Zeng S., Wang H., Zhang S. (2019). Fabrication of multilayered molecularly imprinted membrane for selective recognition and separation of artemisinin. ACS Sustain. Chem. Eng..

[21] Bian W., Zhang R., Chen X., Zhang C., Meng M. (2023). Three-Dimensional Porous PVDF Foam Imprinted Membranes with High Flux and Selectivity toward Artemisinin/Artemether. Molecules.

[22] Wang Y., Ruan H., Zhang J., Huang Y., Guo M., Kong D., Luo J., Yang M. (2023). Recyclable and selective PVDF-based molecularly imprinted membrane combining mussel-inspired biomimetic strategy for dimethomorph elimination. Chem. Eng. J..

[23] Meng M., Li Y., Peng H., Li B., Zhang C., Ren J., Ren Q., Liu Y., Pan J. (2023). Hydrophilic imprinted MnO_2_ nanowires “coating” membrane with ultrahigh adsorption capacity for highly selective separation of Artemisinin/Artemether. Chem. Eng. J..

[24] Zhang X., Wang Y., Xue M., Han Q., Han X. (2023). Enhanced separation properties of polyvinylidene membranes with MnO_2_ nanowire addition. Mater. Today Commun..

[25] Qin L., Zhang W., Cao R. (2023). Hydrophilic MnO_2_ nanowires coating with o-fluoroaniline for electrocatalytic water oxidation. Chin. J. Struct. Chem..

[26] Hong S.K., Kim H., Lee H., Lim G., Cho S.J. (2022). A pore-size tunable superhydrophobic membrane for high-flux membrane distillation. J. Membr. Sci..

[27] Simonin J.P. (2016). On the comparison of pseudo-first order and pseudo-second order rate laws in the modeling of adsorption kinetics. Chem. Eng. J..

[28] Mohamed Idris Z., Hameed B.H., Ye L., Hajizadeh S., Mattiasson B., Din A.T.M. (2020). Amino-functionalised silica-grafted molecularly imprinted polymers for chloramphenicol adsorption. J. Environ. Chem. Eng..

[29] Abu-Alsoud G.F., Hawboldt K.A., Bottaro C.S. (2020). Comparison of four adsorption isotherm models for characterizing molecular recognition of individual phenolic compounds in porous tailor-made molecularly imprinted polymer films. ACS Appl. Mater. Interfaces.

[30] Rabchinskii M.K., Ryzhkov S.A., Besedina N.A., Brzhezinskaya M., Malkov M.N., Stolyarova D.Y., Arutyunyan A.F., Struchkov N.S., Saveliev S.D., Diankin I.D. (2022). Guiding graphene derivatization for covalent immobilization of aptamers. Carbon.

[31] Brzhezinskaya M., Zhivulin E.V. (2022). Controlled modification of polyvinylidene fluoride as a way for carbyne synthesis. Polym. Degrad. Stab..

